# Lachesis Nine Millionth

**Published:** 1887-05

**Authors:** E. W. Berridge

**Affiliations:** London


### LACHESIS NINE-MILLIONTH.

E. W. BERRIDGE, M. D., LONDON.

1886, December 2d, Miss B. wrote that she had had a very bad cold on her chest for eight or nine weeks, which had left behind it a very horrid feeling in her chest, an aching feeling, and a sensation as if she were breathing through a thick blanket; not as if the blanket were at her mouth, but as if it were at the bottom of her throat, just in the little hollow place above the breast-bone (supra sternal fossa). The symptoms are much aggravated if she puts her hand on her chest or on the little hollow place in the neck. For the last few days a little blood has come now and then from the nose on blowing it.

Next day I sent her one dose of the nine-millionth potency of *Lachesis*, which Dr. Fincke had kindly sent me.

1887, January 5th, she wrote that the symptoms went in a week and had not returned.

